#  Langerhans cell histiocytosis presented as bilateral otitis media with effusion, a rare case report

**DOI:** 10.22088/cjim.14.2.401

**Published:** 2023

**Authors:** Soheil Motamed, Maryam Amizadeh, Shahriar Dabiri

**Affiliations:** 1 Department of Otorhinolaryngology, Afzalipour faculty of medicine, Kerman University of Medical Sciences, Kerman, Iran; 2 Student research committee, Kerman University of Medical Sciences, Kerman, Iran; 3 Department of Pathology, Afzalipour faculty of medicine, Kerman University of Medical Sciences, Kerman, Iran

**Keywords:** Langerhans cell histiocytosis, Histiocytosis X, Otitis media with effusion, Children.

## Abstract

**Background:**

Langerhans cell histiocytosis (LCH) or histiocytosis X is considered as a rare disease that may have effect on multiple organs. The initial presentation of LCH is varied. The signs and symptoms of otologic histiocytosis can be the same as the acute or chronic infectious ear diseases. Definitive diagnosis of LCH is confirmed by biopsy and immunohistochemically staining of S-100 protein and CD1a antigen. Chemotherapy is the main mode of treatment.

**Case presentation:**

In this report, we explained the clinical manifestation, diagnosis and treatment of a case of 15 month-old girl with diagnosed of LCH that initially presented with otitis media with effusion (OME).

**Conclusion:**

LCH is a rare disease that presented with variable sign and symptoms and have an effect on multiple organs. LCH should be regarded in cases with recurrent ear infection without response to medical treatments. Moreover, biopsy with IHC is the gold standard of diagnosis and chemotherapy is the main form of treatment.

Langerhans cell histiocytosis (LCH), previously called histiocytosis X, refers to agroup of disorders regarded eosinophilic granuloma. Likewise, Hand-Schüller-Christian disease and Letterer-Siwe disease can be placed in eosinophilic granuloma because of the similarity of their pathologic lesions (1). The presentation of LCH is quite different. In addition, the most common clinical symptom of LCH includes a skin rash or painful bone lesion followed by fever, weight loss, diarrhea, lethargy, chronic cough and dyspnea (2). The reported incidence of otologic manifestations in patients with LCH is 15% to 61%, and they can be considered the first sign of the disease. Also, the most common symptom can be otorrhea, followed by post auricular swelling, hearing loss, and vertigo. The disease may be manifested with perforation of the tympanic membrane, otitis media, otitis externa, fistula between the mastoid and the external canal, and non-tender post-auricular swelling (3, 4). The diagnosis is established by biopsy, besides, microscopic samples including sheets of histiocytes with a variable number of plasma cells, eosinophils, polymorph nuclear leukocytes and multinucleated cells can be observed in biopsy. A definitive diagnosis of LCH is usually made on the basis of immunohistochemistry and occasionally electron microscopic studies (5). In this study, the clinical manifestation, diagnosis and treatment management of a case of Langerhans cell histiocytosis that initially presented and treated with otitis media with effusion (OME) was described.

## Case Presentation

 A 15- month old girl referred to to our otolaryngology department of Shafa Hospital of Kerman University of medical sciences, Kerman, Iran with a history of 6 months bilateral otitis media with effusion and no response to medical treatment was received. She had completed three courses of antibiotics for recurrent bilateral otitis media with effusion (OME). 

**Figure 1:**
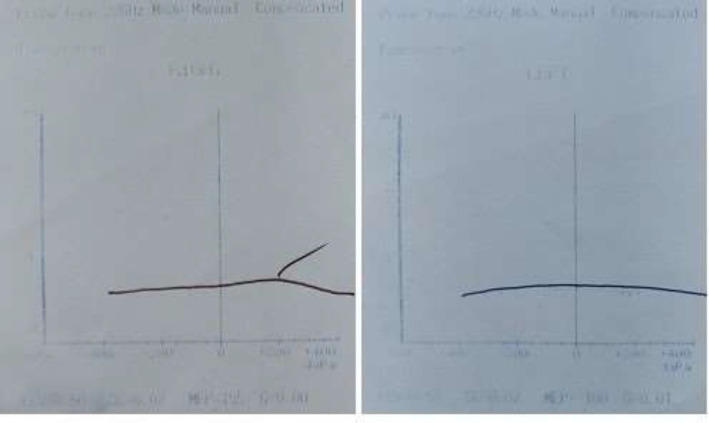
Tympanometry of both ears of the patient

Otomicroscopic examination revealed not any supportive or bloody discharge from both ears. On examination, the child was not in respiratory distress and the facial nerve was intact. Her hematologic testing revealed white blood count 12.8 x109/uL, Hb 12.7 (g/dl), platelet count 479 x109/uL and erythrocyte sedimentation rate (ESR) 11mm. Tympanometry of both ears was B Type with normal ear canal volume (ECV) that was suggestive for OME (figure 1). 

**Figure 2:**
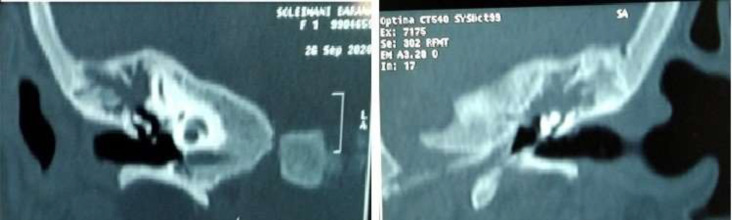
The High Resolution computed tomography (HRCT) of temporal showed bilateral effusion of middle ear

**Figure 3:**
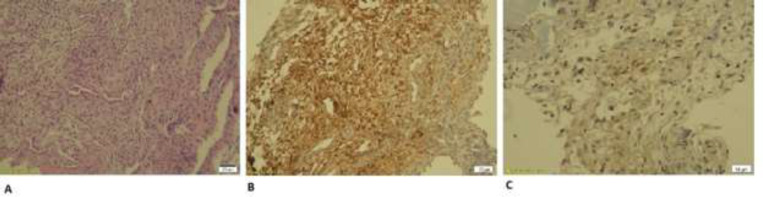
A. hematoxylin and eosin stain demonstrating multifocal mononuclear cells infiltrates as histiocytes (x 100)

**Figure 4:**
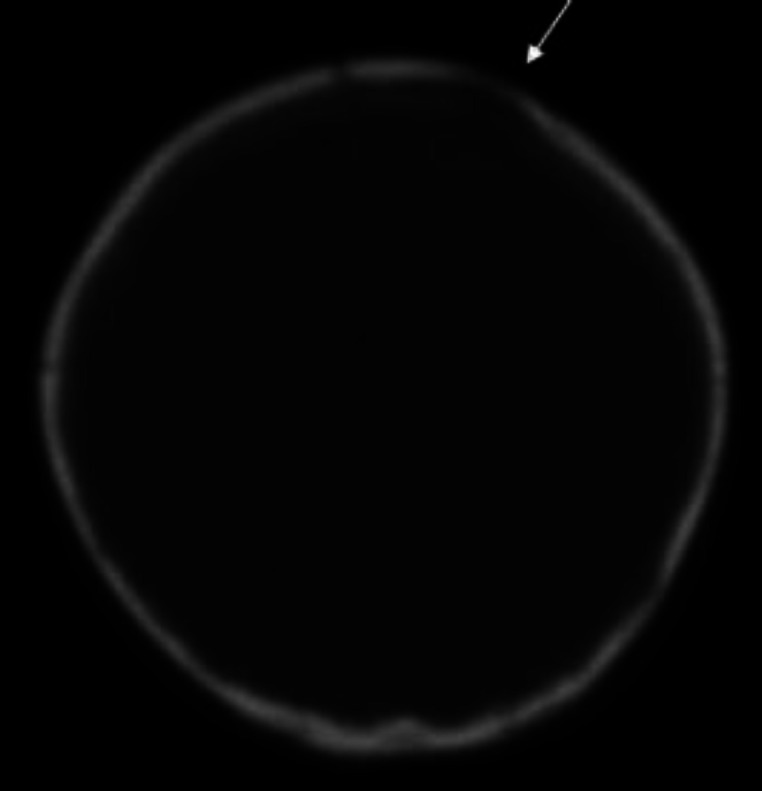
Spiral brain CT scan of a patient: arrow showed a faint lytic lesion in left frontal bone without sclerotic margin.

In addition, the auditory brainstem response (ABR) of the patient showed bilateral mild hearing loss that was in consistent with OME. The High Resolution computed tomography (HRCT) of temporal revealed bilateral effusion of middle ear without destruction of cortical temporal bone and inner ear appeared normal (figure 2) with probable OME diagnosis, the myringotomy and ventilation tube insertion was planned for her; but because of the lack of any effusion from ear in myringotomy operation, biopsy was taken from the right mastoid by post auricular incision under general anesthesia for definite diagnosis. Histopathological examination showed multifocal mononuclear cells infiltrates as histiocytes, a few lymphocytes, fibroblasts and scattered eosinophils. Immunohistochemically (IHC) staining was highly positive for S100 and weakly positive for CD1a. It means that Langerhans histiocytosis is confirmed (figure 3). In further work up for her, no hepatomegaly, lymphadenopathy, exophthalmos and mediastinal mass on physical exam and abdominal sonography were observed on this child. However, in spiral brain CT scan, a faint lytic lesion in left frontal bone without sclerotic margin was seen (figure 4). 

The case who referred to the pediatric hemato-oncologist for therapy and treatment received intravenous vinblastine, MTX and lekuverin with standard dose and during 2 years’ follow-up, had no evidence of any recurrence or metastasis. The audiometry of patient became a type after chemotherapy.

B: Immunohistochemically stain (IHC) with S-100 positivity (x100)

C: Immunohistochemically stain (IHC) stain with CD1a positivity (x200)

## Discussion

The LCH is a group of diseases that can affect any organ or system. Its manifestations range from isolated bone lesions to multisystem disease. Otologic manifestations may include otorrhea from ears, post auricular inflammation, vertigo and subjective hearing loss. And also otitis media, otitis externa with or without granulation tissue, soft tissue masses and osteolytic lesions of the temporal bone are included (6). In our case, the baby girl suffering irritability and ear manipulation for 6 months was primarily treated with otitis media diagnosis but no result was acquired. CT scan is the investigation of choice to detect bony erosion in LCH while MRI is more useful and can depict the soft tissue and intracranial extension (7). In bilateral temporal bone CT scan of the presented case, no bony destruction was shown and MRI was not conducted either. Diagnosis confirmed histologically by tissue biopsy. LCH consists of collection of histiocytes, eosinophils, lymphocytes with few numbers of neutrophils, giant cells and plasma cells. Electron microscopy reveals the presence of Birbeck granules. It’s noteworthy that LCH can be confirmed via immunohistochemical detection of S-100 and CD1 antigen (5, 8). In this case, tissue from bilateral mastoid through post auricular incision was extracted, histopathology revealed histiocytes, a few lymphocytes, fibroblasts and scattered eosinophils, and the diagnosis of LCH was confirmed by positive IHC staining for S-100 protein and CD1 antigen. Treatment protocol is varied among affected individuals. Actually, it is based on the extent of disease at the time of diagnosis. The modalities for LCH treatment include surgery, chemotherapy, radiotherapy, local steroids injection or a combination of them. The localized form of LCH as an isolated bone lesion usually amenable with minimal treatment that is only biopsy or curettage. Treatment for multifocal/multisystem LCH can be done with systemic therapy. Most common drugs used for treatment can be corticosteroids, vinca alkaloids, mercaptopurine, methotrexate, and etoposide (VP16). Histiocyte Society established that combination treatment with vinblastine and prednisolone are effective with minimal side effects; and are the standard therapy for multisystem LCH (9-11). 

Period of treatment be different between 6 weeks to 2 years due to the severity of the tumor. Prognosis of LCH is variable from a rapid fatal to spontaneous resolution. This illness would be shown under than 2 years. Multiple system involvement and presence of cervical lymph nodes are regarded as poor prognostic factors (8). LCH is a rare disease that may have an effect on multiple organs. It’s presented with variable sign and symptoms. LCH should be regarded in cases with recurrent ear infection without response to medical treatments. Moreover, biopsy with IHC is the gold standard of diagnosis and chemotherapy is the main form of treatment. 
